# A Wall-Associated Kinase Gene *CaWAKL20* From Pepper Negatively Modulates Plant Thermotolerance by Reducing the Expression of ABA-Responsive Genes

**DOI:** 10.3389/fpls.2019.00591

**Published:** 2019-05-14

**Authors:** Hu Wang, Huanhuan Niu, Minmin Liang, Yufei Zhai, Wei Huang, Qin Ding, Yu Du, Minghui Lu

**Affiliations:** College of Horticulture, Northwest A&F University, Yangling, China

**Keywords:** pepper, heat stress, wall-associated receptor-like protein kinase, abscisic acid, H2O2

## Abstract

Heat stress has become a major threat to crop production due to global warming; however, the mechanisms underlying plant high-temperature sensing are not well known. In plants, the membrane-anchored receptor-like kinases (RLKs) relay environmental signals into the cytoplasm. In a previous study, we isolated a wall-associated RLK-like (WAKL) gene *CaWAKL20* from pepper (*Capsicum annuum* L.). Here, the amino acid sequence of CaWAKL20 was characterized and found to consist of conserved domains of WAK/WAKL family, including an extracellular region containing a GUB-WAK binding domain and a degenerated EGF2-like domain; a transmembrane region; and an intercellular region with an STKc catalytic domain. Moreover, *CaWAKL20* transcription was inhibited by heat stress, whereas it was induced by both ABA and H_2_O_2_ treatments. Silencing of *CaWAKL20* enhanced pepper thermotolerance, while overexpression decreased Arabidopsis thermotolerance. Additionally, Arabidopsis lines overexpressing *CaWAKL20* showed less sensitivity to ABA during seed germination and root growth. Finally, the survival rate of Arabidopsis seedlings under heat stress treatment was enhanced by ABA pre-treatment, while it was compromised by the overexpression of *CaWAKL20*. Furthermore, the heat-induced expression of several ABA-responsive genes and some key regulator genes for thermotolerance was decreased in Arabidopsis *CaWAKL20-*overexpression lines. These results suggest that *CaWAKL20* negatively modulates plant thermotolerance by reducing the expression of ABA-responsive genes, laying a foundation for further investigation into the functional mechanisms of WAKs/WAKLs in plants undergoing environmental stresses.

## Introduction

Plants frequently face adverse environmental conditions, including extreme temperature, drought, salinity, and flood that can negatively affect plant growth, development, production, and even survival. As global warming worsens, heat waves are occurring with increased frequency and longer duration, especially in lower latitude regions (IPCC, 2014), and the resultant heat stress is becoming an increasingly significant problem for crop production and world food security (Ohama et al., 2017).

Heat stress can cause protein denaturation and aggregation, reduce cellular functions, and even result in cell death (Driedonks et al., 2015). To defend against these negative effects, as sessile organisms, plants have developed a conserved heat stress response (HSR) system to induce the expression of stress-related genes (Mittler et al., 2012). However, relatively little is known about how plants sense high temperature, which then hinders the establishment of systems that protect against heat stress in crop breeding and/or cultivation.

Under heat stress, the HSR can be triggered by increased fluidity of the plasma membrane (Sangwan et al., 2002) or accumulation of unfolded proteins (Rütgers et al., 2017). The plasma membrane is suggested to be a primary heat sensor in plants (Hofmann, 2009), and changes in cytomembrane fluidity can be sensed by integral membrane proteins, such as ion channels and/or transporters, as well as membrane-anchored receptor-like kinases (RLKs) (Zhu, 2016). While the involvement of membrane calcium channels in plant HSR has been confirmed (Saidi et al., 2009; Finka et al., 2012), the contribution of RLKs to plant thermotolerance is poorly understood.

Plant RLKs are classed as transmembrane proteins containing an N-terminal extracellular domain and a C-terminal intracellular kinase domain. Receptor-like kinases are thought to participate in a diverse range of life processes including growth, development, hormone signalings, plant-microbe interactions, and responses to environmental stimuli (Shiu and Bleecker, 2001; Ye et al., 2017).

As an RLK subfamily, wall-associated receptor kinase (WAK) and WAK-like (WAKL) are characterized by the presence of an extracellular epidermal growth factor (EGF)-like domain. These WAKs/WAKLs physically link the cell wall to the plasma membrane, directly transmitting extracellular signals into the cytoplasm to regulate cell growth and stress responses (Anderson et al., 2001; Kohorn, 2016). Zhang et al. (2017) even suggested that “ZmWAK functions as a hub in the trade-off between maize growth and defense.”

Since firstly reported in Arabidopsis (He et al., 1998), the contributions of WAKs/WAKLs to plant immunity have been widely studied, including AtWAKL22 in Arabidopsis resistance to *Fusarium oxysporum* (Diener and Ausubel, 2005), *SlWAK1* in tomato resistance to *Pst* (Rosli et al., 2013), *OsWAKs* in rice resistance to fungal blast and bacterial blight disease (Li et al., 2009; Delteil et al., 2016; Harkenrider et al., 2016), *ZmWAKs* in maize resistance to northern corn leaf blight and head-smut disease (Hurni et al., 2015; Zhang et al., 2017; Yang et al., 2018), and *TaWAKL4* in wheat resistance to *Zymoseptoria tritici* (Saintenac et al., 2018).

An increasing number of studies have reported on the involvement of WAKs/WAKLs in plant tolerance to abiotic stresses. For example, Sivaguru et al. (2003) reported that overexpression of *AtWAK1* enhanced Arabidopsis tolerance to heavy metal stress, whereas Hou et al. (2005) found that T-DNA insertion in the *AtWAKL4* promoter increased Arabidopsis hypersensitivity to salt stress. Furthermore, Xia et al. (2018) suggested that, when subjected to excess Cu, OsWAK11 can modify cell wall properties to retain Cu at the cell wall. However, it remains to be elucidated whether *WAK/WAKL* genes are involved in plant thermotolerance.

In our previous work, we isolated a heat-responsive gene *Capana12g000852* from the pepper plant. Due to its high amino acid sequence homology with Arabidopsis *AtWAKL20*, *Capana12g000852* was renamed *CaWAKL20*. In this study, we show that silencing the *CaWAKL20* gene enhanced pepper tolerance to heat stress, whereas overexpressing *CaWAKL20* weakened Arabidopsis thermotolerance. In addition, Arabidopsis lines overexpressing *CaWAKL20* exhibited reduced sensitivity to abscisic acid (ABA) during seed germination and seedling growth. We also show that, under heat stress treatment, the induced expression of some ABA-responsive genes was reduced in *CaWAKL20*-overexpressing Arabidopsis lines. Our study may contribute to the understanding of the molecular mechanisms of plant tolerance against heat stress.

## Materials and Methods

### Plant Materials and Growth Conditions

The pepper R9 thermotolerant line (introduced from the World-Asia Vegetable Research and Development Center, PP0042-51) and Arabidopsis ecotype Col-0 were used in this study. Plant materials were grown in a chamber under 200 μmol⋅m^–2^⋅s^–1^ illumination intensity, a 16 h day/8 h night regime, and 70% relative humidity. The temperature was set to 26°C/20°C (day/night) for pepper and 22°C/18°C for Arabidopsis.

### Analysis of the Deduced CaWAKL20 Amino Acid Sequence

The CaWAKL20 (Capana12g000852) amino acid sequence was downloaded from the PGD database^1^, and those of Arabidopsis WAKs/WAKLs were obtained from TAIR (The Arabidopsis Information Resource)^2^ (Verica and He, 2002). The conserved domains in the CaWAKL20 amino acid sequence were identified using the online SMART tool (Simple Modular Architecture Research Tool)^3^. Full-length CaWAKL20 and AtWAKs/WAKLs were aligned using the online Clustal Omega program^4^, and the phylogenetic tree was generated by MEGA 6 software using the neighbor-joining method, based on the *p*-distance substitution model, and with 1000 bootstrap replicates (Tamura et al., 2013).

Arabidopsis AtWAKL20 was used to predict the CaWAKL20 protein-protein interaction network in the STRING interaction database (Search Tool for the Retrieval of Interacting Genes/Proteins)^5^ with a 0.400 confidence score. Finally, the output was imported into Cytoscape_v3.4.0 (National Institute of General Medical Sciences, MD, United States) to generate the network map.

### Subcellular Localization of CaWAKL20

The *CaWAKL20* coding sequence (CDS) without a stop codon was amplified from the pepper line R9 using the GFP-CaWAKL20-F and GFP-CaWAKL20-R primer pair (Supplementary Table S1); the PCR product was then cloned into a GFP-tagged pBI221 transient expression vector. The empty vector was used as the control. Particle bombardment was used to transfect plasmid into onion epidermal cells, which were then incubated for 24 h at 28°C in the dark. The GFP signal was visualized using A1R confocal laser scanning microscopy (Nikon, Tokyo, Japan). For plasmolysis analysis, the transformed onion epidermal cells were treated in 0.8 M mannitol solution for 15 min before observation of GFP expression (Genovesi et al., 2008).

### Virus-Induced Gene Silencing (VIGS) of *CaWAKL*20

To generate gene-silenced plants using VIGS, a conserved 346 bp fragment in the *CaWAKL20* CDS was amplified from the pepper line R9 with the TRV2-CaWAKL20-F and TRV2-CaWAKL20-R primer pair (Supplementary Table S1), and the PCR product was inserted into a pMD19-T vector (Takara, Dalian, China). After being digested with *Eco*R I and *Bam*H I, the resultant *CaWAKL20* gene fragment was cloned into the pTRV2 virus expression vector to generate the TRV2:*CaWAKL20* gene-silencing vector. The empty vector was used as the control and was referred to as TRV2:00, and the TRV2:*CaPDS* (phytoene desaturase gene) vector was used as the marker for successful gene silencing. The *Agrobacterium tumefaciens* strain GV3101 containing TRV2:*CaWAKL20*, TRV2:00, or TRV2:*CaPDS* was injected into the cotyledons of the pepper line for gene silencing. When the photo-bleaching phenotype was clearly observable in newly grown leaves of plants transformed with TRV2:*CaPDS*, the transcription of TRV1 and TRV2 in plants expressing TRV2:00 and TRV2:*CaWAKL20* was assessed by semi-quantitative PCR using the TRV1-TL-F/TRV1-TL-R and TRV2-Coat P-F/TRV2-Coat P-R primer pairs, respectively (Supplementary Table S1, Tsaballa et al., 2011). The efficiency of silencing of *CaWAKL20* expression in plants expressing TRV2:*CaWAKL20* was assessed by qRT-PCR using the qCaWAKL20-F and qCaWAKL20-R primer pair (Supplementary Table S1).

### Generation of Arabidopsis Lines Overexpressing *CaWAKL*20

The complete *CaWAKL20* CDS was amplified from the R9 line using the CaWAKL20-F and CaWAKL20-R primer pair (Supplementary Table S1), and the amplification product was cloned into the pVBG2307 plant binary expression vector under the control of the CaMV35S promoter. Using *A. tumefaciens* strain GV3101, the 35S::*CaWAKL20* vector was transformed into Arabidopsis ecotype Col-0 by the floral dip method (Clough and Bent, 1998). Transgenic plants were screened by adding kanamycin into the MS culture medium, and the T3 generation was then used for subsequent experiments.

### Experimental Treatments and Collection of Samples

For tissue-specific expression analysis of *CaWAKL20*, samples of seeds, roots, stems, young leaves, flower buds, and young fruits were collected from pepper plants grown under normal conditions.

For analysis of *CaWAKL20* expression under stress treatments, pepper seedlings at the six-leaf stage were either incubated at 45°C for heat stress treatment, sprayed evenly with a 0.1 mM ABA solution for ABA treatment, or immersed with roots in a 100 mM H_2_O_2_ solution for H_2_O_2_ treatment. The young leaves were collected at 0, 1, 3, 6, 12, and 24 h post-treatments, and all the samples were immediately frozen in liquid nitrogen and stored at −80°C for RNA extraction.

To assess the efficiency of silencing of *CaWAKL20* expression, pepper seedlings containing TRV2:*CaWAKL20* and TRV2:00 were incubated at 45°C for 1 h. To measure the thermotolerance of *CaWAKL20-*silenced plants, pepper seedlings containing TRV2:*CaWAKL20* and TRV2:00 were incubated at 45°C for 10 h and then allowed to recover for 24 h. The pepper leaves were sampled at the end of both treatments and either stored at −80 °C for gene expression analysis or used immediately to determine malondialdehyde (MDA) content and the maximal photochemical efficiency of PSII (*Fv*/*Fm*).

For thermotolerance evaluation of plants overexpressing *CaWAKL20*, transgenic Arabidopsis lines overexpressing *CaWAKL20* (*CaWAKL20*-OE) or containing the empty vector (EV) were used. Ten days old Arabidopsis seedlings on MS plates were immersed in a water bath at 45°C for 50 min and then allowed to recover for 2 days at 22°C. Three weeks old Arabidopsis seedlings in pots were incubated at 45°C for 12 h and allowed to recover for 7 days at 22°C. The survival rates of Arabidopsis seedlings were calculated at the end of both treatments, and leaves from seedlings in pot were sampled at 3 h post-heat stress treatment for gene expression analysis. Three weeks old Arabidopsis seedlings in pots were treated at 45°C for 12 h and then collected for the assessment of H_2_O_2_ accumulation.

To assess the effects of *CaWAKL20* overexpression on Arabidopsis sensitivity to ABA, seeds of *CaWAKL20*-OE and EV lines were germinated on MS plates containing 0, 0.75, and 1 μM ABA. The germination rate, green cotyledon rate, and root length were determined at 4, 9, and 11 days after treatment, respectively. All experiments were performed with three biological replicates.

To evaluate the effects of exogenous ABA on the thermotolerance of plants overexpressing *CaWAKL20*, 10 days old seedlings of Arabidopsis lines of EV, OE2, and OE14 were transferred to MS medium with or without 5 μM ABA (Wang et al., 2017). After pre-treatment with ABA for 2 days, the plates were immersed in a water bath at 45°C for 50 min and then allowed to recover for 2 or 3 days at 22°C. The rate of green seedlings was calculated at different time points after treatments.

### Determination of MDA Content, PSII *Fv/Fm* and H_2_O_2_ Accumulation

The MDA content of the pepper leaves was measured using the thiobarbituric acid assay (Dhindsa et al., 1981). The PSII *Fv*/*Fm* of pepper leaves was determined using the FluorCam7 fluorescence imaging system (EcoTech, China). The assessment of H_2_O_2_ accumulation in Arabidopsis seedlings was performed using the diaminobenzidine (DAB) staining method (Dang et al., 2013). All experiments were performed with three biological replicates.

### Total RNA Extraction, cDNA Synthesis, and qRT-PCR Analysis

Total RNA was extracted from the leaves of pepper and Arabidopsis plants using the Trizol^®^ kit (Invitrogen, Carlsbad, CA, United States), and the residual genomic DNA was digested with RNase-free DNase I (Promega, Madison, WI, United States). First-strand cDNA synthesis was performed using the PrimeScript^TM^ Kit (TaKaRa, Tokyo, Japan) according to the manufacturer’s instructions. Primer pairs were designed using Primer-BLAST in NCBI^6^ (Supplementary Table S1), and qRT-PCR was performed using SYBR^®^ Premix Ex Taq^TM^ II (TaKaRa). Relative gene expression levels were analyzed according to the 2^–ΔΔCT^ method (Livak and Schmittgen, 2001), in which *CaUBI3* and *AtActin2* were used as internal controls for pepper and Arabidopsis, respectively. Significance tests for differences in gene expression between control and stress treatments were performed using the Student’s *t*-test method at the 0.05 and 0.01 significance levels.

## Results

### Analysis of the Deduced *CaWAKL20* Amino Acid Sequence

Domains that are conserved in the WAK/WAKL protein family were identified in the amino acid sequence of Capana12g000852 (Supplementary Figures S1A,B) using the SMART online tool. The conserved domains included an extracellular region containing a signal peptide and a GUB-WAK binding domain (galacturonan-binding, pfam13947) at the N-terminus, a transmembrane region, and an intercellular region with a catalytic STKc domain (Serine/Threonine kinase, cd14066) at the C-terminus. The EGF domain, a marker for the WAK subfamily that distinguishes it from others in RLKs (Anderson et al., 2001), was absent in Capana12g000852 (Supplementary Figure S1A); however, a degenerated EGF2-like domain (Prosite: PS01187) (Verica et al., 2003) was present (Supplementary Figure S1B), which is also observed for AtWAKL20 (Verica and He, 2002). In addition, Capana12g000852 displayed a closer phylogenetic relationship with AtWAKL20 than with the other 25 WAK/WAKL members found in Arabidopsis (Verica and He, 2002; Supplementary Figure S1C), and was therefore renamed CaWAKL20.

To further understand its functional patterns, the CaWAKL20 protein-protein interaction network was predicted using the STRING online tool based on Arabidopsis interologs. Ten CaWAKL20 interaction partners were identified, including a WAK family member (WAKL7), a zinc ion binding protein, and eight protein phosphatase 2C (PP2C) family members, i.e., ABI2 (ABA Insensitive 2), AHG1 (ABA-Hypersensitive Germination 1), APD9 (Arabidopsis PP2C clade D9), EGR2 (E Growth-Regulating 2), HAI1 (Highly ABA-Induced PP2C gene 1), and PP2C74, 76, and 80 (Supplementary Figure S1D).

### Subcellular Localization of CaWAKL20

**FIGURE 1:**
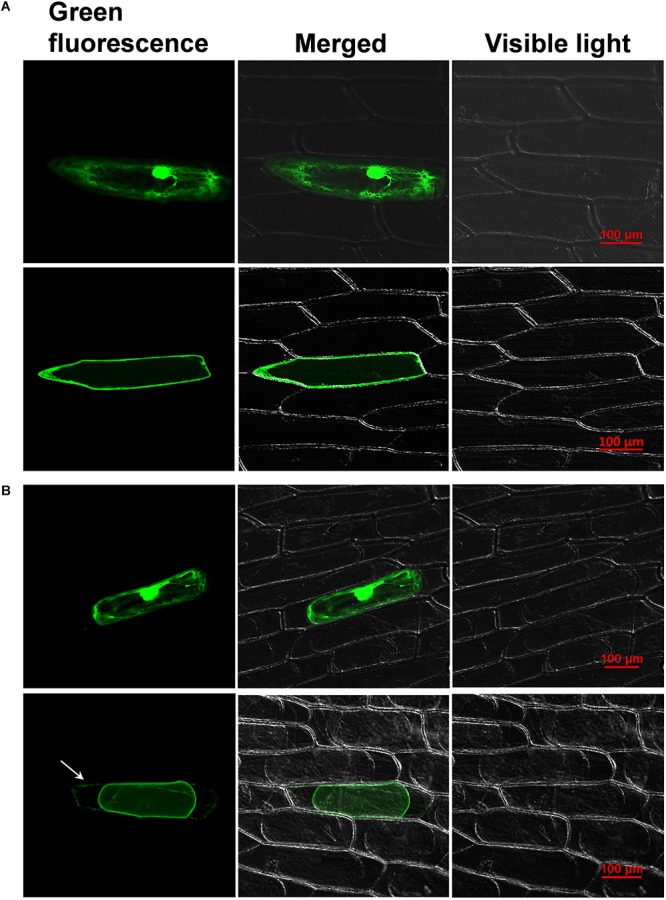
Fluorescence microscopy images of **(A)** normal and **(B)** plasmolyzed onion epidermal cells transiently expressing either free green fluorescent protein (GFP) (top) or 35S: CaWAKL20-GFP fusion proteins (bottom). The arrow shows the GFP signal at the cell wall. Scale bar = 100 μm.

To confirm the WAK/WAKL classification of CaWAKL20, the GFP-tagged *CaWAKL20* CDS was transiently expressed in onion epidermal cells under the control of the CaMV35S promoter. In the cells transformed with the empty control vector, the GFP signal was distributed throughout the entire cell. In contrast, green fluorescence was detected along the edge of the cells transformed with the CaWAKL20-GFP fusion vector (Figure 1A).

To further test the membrane- and cell wall-binding properties of CaWAKL20, the onion epidermal cells transformed with CaWAKL20 were cultured in mannitol for plasmolysis. In plasmolyzed cells, the green fluorescence of the CaWAKL20-GFP protein was observed both in the plasma membrane and the cell wall (Figure 1B). This suggests that CaWAKL20 was localized at the plasma membrane and linked to the cell wall.

### Expression of *CaWAKL20* in Different Pepper Plant Tissues and Under Heat Stress, ABA, and H_2_O_2_ Treatments

**FIGURE 2:**
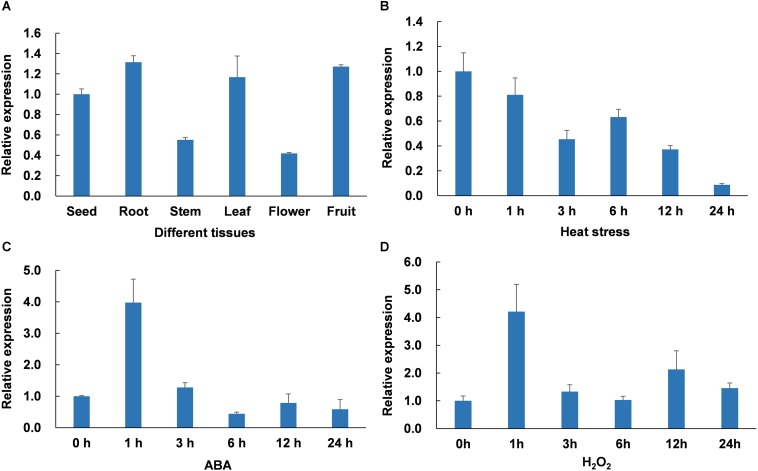
*CaWAKL20* expression patterns **(A)** in different pepper tissues, under **(B)** heat stress induction at 45°C, **(C)** 0.1 mM ABA spraying, and **(D)** 100 mM H_2_O_2_ root-immersing. The expression of *CaWAKL20* was normalized to the ubiquitin-conjugating protein-coding gene *CaUBI3*. The experiment was conducted with three biological replicates, and each replicate contained five pepper seedlings; error bars represent standard deviations for three biological replicates.

To explore the expression patterns of *CaWAKL20* in different pepper plant tissues, the roots, stems, leaves, flowers, and fruits of plants grown under normal conditions were sampled from the R9 thermotolerant line, and qRT-PCR was performed using a *CaWAKL20*-specific primer pair. The results indicated that *CaWAKL20* was variably expressed in different tissues, and the expression levels were clearly lower in the stems and flowers than in the other tissues (Figure 2A).

The CaWAKL20 protein was predicted to interact with several proteins related to ABA (Supplementary Figure S1D), a major phytohormone essential for plant responses to a wide range of stresses (Vishwakarma et al., 2017). In addition, reactive oxygen species (ROS) also play a key role in plants’ acclimation to abiotic stresses, including heat (Suzuki and Katano, 2018). Therefore, to elucidate the response of *CaWAKL20* to heat stress, ABA and ROS treatments, *CaWAKL20* expression was analyzed following incubation at 45°C and exposure to 0.1 mM ABA and 100 mM H_2_O_2_, respectively. The results showed that *CaWAKL20* transcription responded to all treatments. For heat stress, *CaWAKL20* expression decreased continuously during the whole treatment (Figure 2B), while with both ABA and H_2_O_2_ treatments, *CaWAKL20* expression increased at 1 h, and then declined rapidly (Figures 2C,D).

### Thermotolerance Was Enhanced in *CaWAKL20*-Silenced Pepper Seedlings

To understand the role of *CaWAKL20* in pepper thermotolerance, *CaWAKL20* was silenced in the thermotolerant R9 pepper line. The fragment of *CaWAKL20* used for VIGS was predicted to have no off-target potential (by the VIGS tool in SGN^7^) and was therefore presumed to be specific for the *CaWAKL20* gene. Approximately 1 month after injection for VIGS, the bleaching was clearly observed in the leaves of positive control pepper seedlings containing TRV2:*CaPDS*; however, no visible phenotype was observed in leaves with either TRV2:00 or TRV2:*CaWAKL20* (Supplementary Figure S2A). Both the RNA1 segment in TRV1 (GenBank: AF406990) and the coat protein in TRV2 (GenBank: AF406991) were almost equally expressed in all virus-infected pepper plants (Supplementary Figure S2B). Compared to plants with TRV2:00, the expression of the *CaWAKL20* gene was silenced to approximately 70% in the plants with TRV2:*CaWAKL20*, which became more evident after the heat stress of 45°C for 1 h (Supplementary Figure S2C).

**FIGURE 3:**
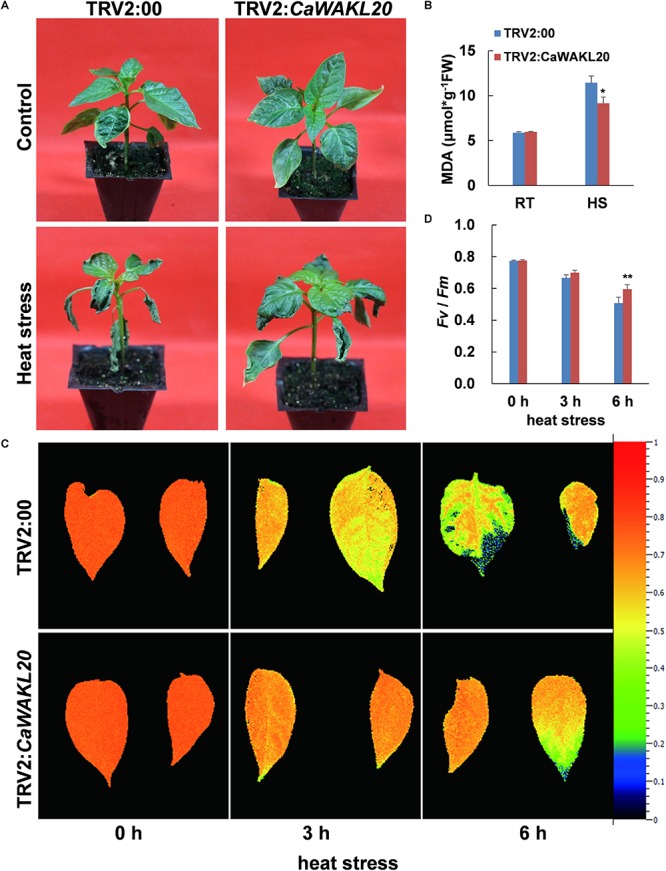
Thermotolerance of *CaWAKL20*-silenced pepper seedlings. **(A)** Performance of intact plants, **(B)** MDA contents in leaves, and **(C,D)** the maximal photochemical efficiency of PSII (*Fv*/*Fm*) after heat stress. RT, room temperature; HS, heat stress incubation at 45°C for 10 h, and then recovery for 24 h. Error bars represent standard deviations for three biological replicates; * and ** indicate significant differences at the 0.05 and 0.01 levels, respectively.

After exposure to heat stress at 45°C for 10 h and recovery for 24 h under normal conditions, the leaves of the pepper plants containing TRV2:00 were severely parched, while those with TRV2:*CaWAKL20* resumed growing (Figure 3A). Under heat stress treatment, the MDA content increased from 5.88 to 11.44 μmol*g^–1^ fresh weight (FW) in leaves of plants with TRV2:*CaWAKL20*, whereas for plants with TRV2:00 the MDA content in the leaves increased from 5.98 to 9.15 μmol*g^–1^FW. After heat treatment, the MDA content was significantly higher in the leaves of plants with TRV2:00 than those with TRV2:*CaWAKL20* (*p* < 0.05) (Figure 3B). Furthermore, with prolonged heat stress, the PSII *Fv*/*Fm* declined continuously in the leaves of plants transformed with both TRV2:*CaWAKL20* and TRV2:00; at the end of treatment, the *Fv*/*Fm* value was clearly higher in the former (0.60) than that in the latter (0.51) (*p* < 0.01) (Figures 3C,D).

### Thermotolerance Was Reduced in *CaWAKL20*-Overexpressing Arabidopsis Lines

To further understand the role of *CaWAKL20* in plant thermotolerance, Arabidopsis lines overexpressing *CaWAKL20* (OE) were generated, with OE2 and OE14 being selected and used for further studies (Supplementary Figures S2D,E).

**FIGURE 4:**
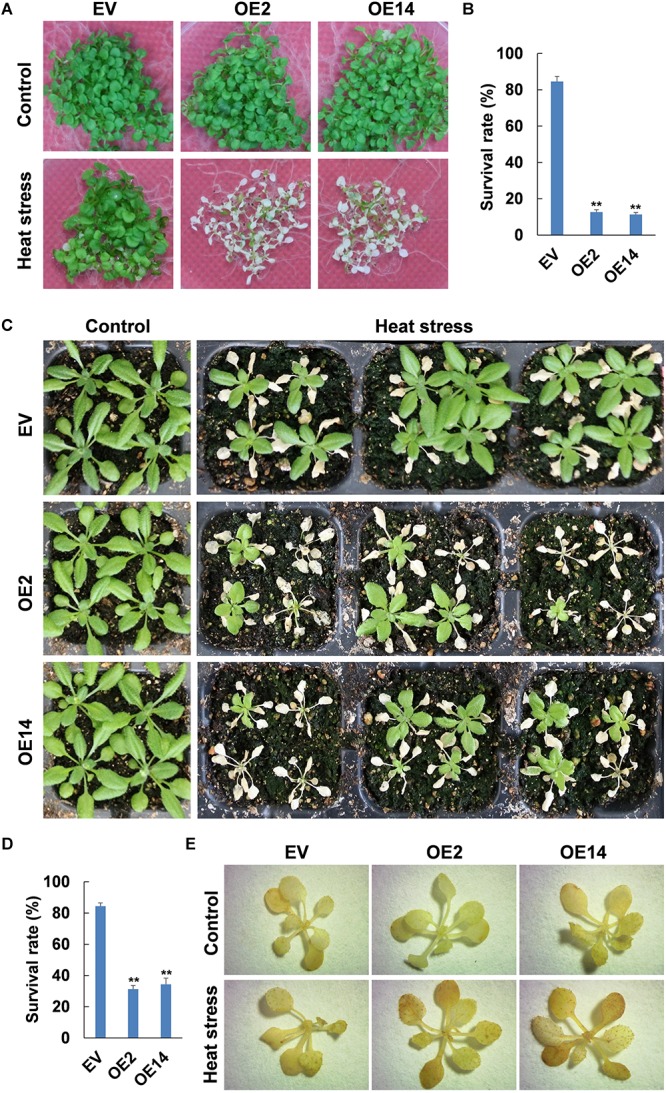
Thermotolerance of *CaWAKL20*-overexpressing Arabidopsis lines. **(A,C)** Phenotypes, **(B,D)** survival rates, and **(E)** H_2_O_2_ accumulation of Arabidopsis seedlings grown **(A,B)** on plates or **(C–E)** in pots under heat stress (45°C in a water bath) **(A,B)** for 50 min, and then recovery for 2 days or for **(C–E)** 12 h and then **(C,D)** recovery for 7 days. OE2 and OE14, transgenic Arabidopsis lines overexpressing *CaWAKL20*; EV, control trangenic Arabidopsis line with the empty vector. Error bars represent standard deviations for three replicates, and each replicate contained five plates **(A,B)** or 12 Arabidopsis seedlings **(C–E)**; ** indicates a significant difference at the 0.01 level.

For 10 days old Arabidopsis seedlings on MS plates, after induction of heat stress at 45°C for 50 min followed by recovery for 2 days, the survival rate was higher than 80% in the EV line compared to only approximately 12% for seedlings in either *CaWAKL20-*OE line (Figures 4A,B). For 3 weeks old Arabidopsis in pots, after induction of heat stress at 45°C for 12 h and recovery for 7 days, the survival rate was higher than 80% in the EV line compared to only 31% in the OE2 line and 34% in the OE14 line (Figures 4C,D). Meanwhile, the seedlings from the *CaWAKL20-*OE lines that survived had fewer green leaves than those from the EV line. After heat stress treatment at 45°C for 12 h, the level of DAB staining increased in all Arabidopsis seedlings. Compared with that of the EV line, the staining intensity in the seedlings of both OE2 and OE14 were obviously raised (Figure 4E), suggesting the higher levels of H_2_O_2_ accumulation in *CaWAKL20-*OE lines.

### ABA Sensitivity Decreased in Arabidopsis *CaWAKL20*-OE Lines

**FIGURE 5:**
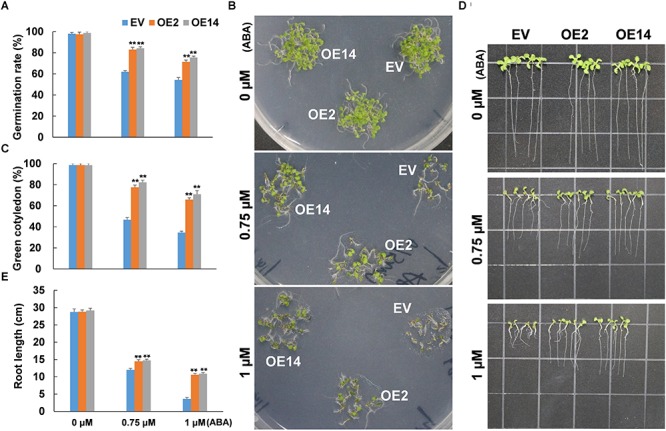
The sensitivity to ABA in terms of seed germination and seedling growth in *CaWAKL20*-overexpressing Arabidopsis lines. **(A)** Seed germination rate, **(B,C)** green cotyledon rate, and **(D,E)** root length of Arabidopsis at the **(A)** 4, **(B,C)** 9, and **(D,E)** 11 days after treatments with 0, 0.75, and 1 μM ABA, respectively. OE2 and OE14, Arabidopsis transgenic lines overexpressing *CaWAKL20*; EV, control Arabidopsis transgenic line containing the empty vector. Error bars represent standard deviations for three replicates, and each replicate contained **(A–C)** 5 plates or **(D,E)** 12 Arabidopsis seedlings. ** indicates a significant difference at the 0.01 level.

No difference was observed in the seed germination rate between *CaWAKL20*-OE and EV Arabidopsis lines under normal growth conditions. Under ABA treatments, the seed germination rate was reduced, but more so in the EV line than in the *CaWAKL20*-OE lines (Figure 5A). When the treatments were extended to 9 days, the growth of *CaWAKL20*-OE seedlings was clearly better than those of the EV line (Figure 5B). Under 0.75 and 1 μM ABA treatments, the percentage of green cotyledons decreased to 47 and 35%, respectively, in the EV line, but to 77 and 65%, respectively, in the *CaWAKL20-*OE lines (Figure 5C). Furthermore, root growth was suppressed by ABA treatments in both the *CaWAKL20*-OE and EV lines; however, root length was clearly greater in the former than in the latter (Figure 5D). For example, under 1 μM ABA treatment, the root length of the *CaWAKL20*-OE seedlings was approximately twofold greater than that of EV seedlings (Figure 5E).

### ABA-Enhanced Thermotolerance Was Compromised by Overexpression of *CaWAKL20* in Arabidopsis

**FIGURE 6:**
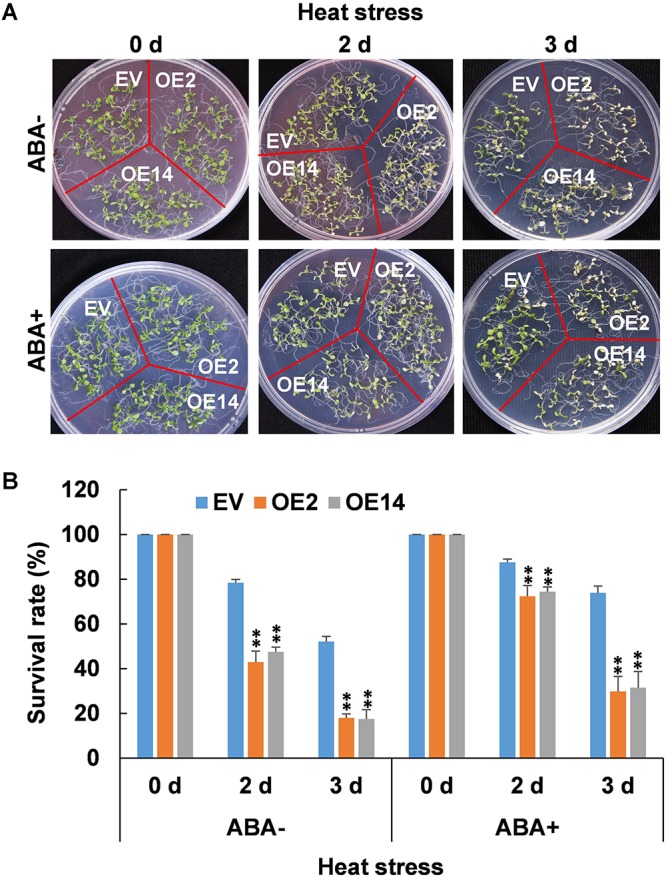
Thermotolerance of *CaWAKL20*-overexpressing Arabidopsis lines under ABA pretreatment. **(A)** Phenotypes and **(B)** survival rates of Arabidopsis seedlings grown on plates under heat stress (45°C in a water bath) for 50 min, and then recovery for 2 or 3 days. OE2 and OE14, transgenic Arabidopsis lines overexpressing *CaWAKL20*; EV, control transgenic Arabidopsis line with the empty vector. Error bars represent standard deviations for three replicates, and each replicate contained five plates; ** indicates a significant difference at the 0.01 level.

To clarify the relationship between ABA treatment and *CaWAKL20* expression in the development of plant thermotolerance, the seedlings of Arabidopsis *CaWAKL20*-OE were pretreated with ABA for 2 days and then exposed to heat stress. The results showed that the heat stress decreased the survival rate of Arabidopsis seedlings of all three lines in both with and without ABA pre-treatment, and the survival rates in OE2 and OE14 were significantly lower than those in EV line in both the tested time points (Figure 6). Compared to those without ABA pre-treatment (ABA-), however, the survival rate of Arabidopsis seedlings under heat stress was enhanced by ABA pre-treatment (ABA+) in all three Arabidopsis lines of EV, OE2, and OE14 (Figure 6A). Furthermore, under the treatment of ABA-, in OE2 and OE14, the survival rate was lower than in EV line by about 35 and 31% after 2 days of heat stress, and about 34 and 35% after 3 days of heat stress, respectively. Under the ABA+ treatment, the survival rate was lower in OE2 and OE14 than in EV line by about 15 and 13% after 2 days of heat stress, and about 44 and 42% after 3 days of heat stress, respectively (Figure 6B).

### Heat Stress-Induced Expression of ABA-Responsive Genes Was Reduced in Arabidopsis *CaWAKL20*-OE Lines

**FIGURE 7:**
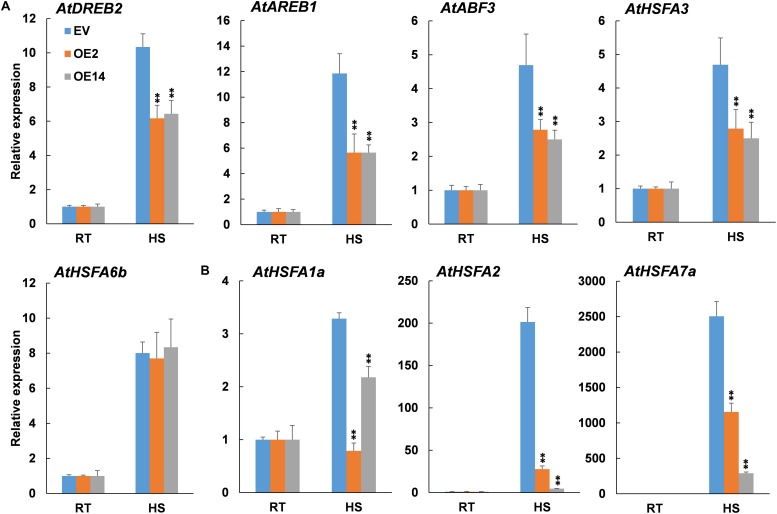
Expression of **(A)** ABA-responsive genes and **(B)** key regulator genes for thermotolerance in *CaWAKL20*-overexpressing Arabidopsis lines after heat stress treatment. RT, room temperature; HS, heat stress induction at 45°C for 3 h. *AtActin2* was used to normalize gene expression, and the expression level under 0 h after treatment was taken as 1.0. Error bars represent standard deviations for three replicates, and each replicateconsisted of 12 seedlings. ** indicates significant difference at the 0.01 level.

To confirm the involvement of ABA in *CaWAKL20*-mediated thermotolerance, we further examined the expression of several heat-related and simultaneously ABA-responsive genes in Arabidopsis EV line and *CaWAKL20*-OE lines under heat stress; the genes included *AREB* (Fujita et al., 2005), *ABF* (Choi et al., 2013), *HSFA6b* (Huang et al., 2016), *DREB*, and *HSFA3* (Schramm et al., 2008). All the genes were induced by heat stress in both the EV line and *CaWAKL20-OE* lines; however, except for *AtHSFA6b*, the expression levels were clearly lower in the *CaWAKL20*-OE lines than in the EV line (Figure 7A).

In addition, the expression of several key regulator genes for plant thermotolerance, *HSFA1a*, *HSFA2*, and *HSFA7a* (Yoshida et al., 2011), was also assessed under heat stress. The results showed that in line with those of ABA-responsive genes, the transcription of all tested thermotolerance-regulator genes was enhanced by heat stress, whereas this enhancement was inhibited by the overexpression of *CaWAKL20* in both OE2 and OE4 (Figure 7B).

## Discussion

In a previous study, we isolated a heat-responsive gene *Capana12g000852* from the pepper plant (data not shown). Further conserved domain analysis identified all the necessary WAK/WAKL domains in the deduced Capana12g000852 amino acid sequence (Supplementary Figures S1A,B). In addition, Capana12g000852 showed a similar domain distribution and closer phylogenetic relationship to Arabidopsis AtWAKL20 (Supplementary Figures S1A,C). From these data, we suggest that *Capana12g000852* is a pepper homolog of *AtWAKL20* and rename it *CaWAKL20*. The *WAK/WAKL* properties of *CaWAKL20* are also supported by its subcellular localization in onion epidermal cells (Figure 1).

Although WAKs/WAKLs participate in plant responses to various biotic and abiotic stresses as a plasma membrane localized receptor-like kinase (Anderson et al., 2001), their roles in thermotolerance are unclear. Here, we showed that *CaWAKL20* expression was downregulated in a manner that was dependent on the duration of the heat stress treatment (Figure 2B). When *CaWAKL20* was silenced, pepper plant thermotolerance was enhanced as evidenced by the smaller increase in MDA content and the lower decline in the *Fv*/*Fm* value, compared to the plants transformed with the empty TRV2:00 vector (Figure 3). In contrast, the Arabidopsis *CaWAKL20*-OE lines displayed reduced thermotolerance in terms of seedling survival rate and ROS accumulation (Figure 4). These data suggest that *CaWAKL20* negatively modulates plant thermotolerance.

The induced expression of WAK/WAKL genes is widely believed to be a requirement for plant survival during pathogen infection or heavy metal stress (He et al., 1998; Sivaguru et al., 2003); however, some contradictory results have also been reported. Harkenrider et al. (2016) found that overexpression of *OsWAK25* increased rice susceptibility to necrotrophic fungal pathogens, although it enhanced rice resistance to hemibiotrophic pathogens. OsWAK14, OsWAK91, and OsWAK92 positively regulate rice resistance to blast fungus, while OsWAK112d functions as a negative regulator (Delteil et al., 2016). When the AtWAKL4 promoter was impaired, Arabidopsis tolerance to K^+^, Na^+^, Cu^2+^, and Zn^2+^ was reduced, but its tolerance to Ni^2+^ was enhanced (Hou et al., 2005). Therefore, WAKs/WAKLs may have differential roles in plant tolerance against biotic and/or abiotic stresses, and/or the roles may be stress type-dependent. As far as *CaWAKL20*, its functional model in the pepper plant response to heat stress remains to be further elucidated.

When contending with adverse environments, plants activate their protective mechanisms that, conversely, often suppress plant growth to focus energy on defending against the stress. Therefore, the ability to switch from growth to defense is crucial for plants’ survival under stressed conditions (Wang and Wang, 2014; Albrecht and Argueso, 2017). Several hubs for tuning plant stress signaling and development have been reported in plants, such as CDPKs (Calcium-Dependent Protein Kinases) (Schulz et al., 2013) and WAKs/WAKLs (Kohorn, 2016). Zhang et al. (2017) found that maize ZmWAK promotes cell growth in the absence of pathogens but switches to a protective role when the maize plant is attacked by *Sporisorium reilianum*. In our study, after heat stress treatment, *CaWAKL20* expression in the pepper thermotolerant line declined continuously (Figure 2B); similarly, Giarola et al. (2016) also observed that *CpWAK1* transcription in *Craterostigma plantagineum*, a plant species with tolerance to extreme desiccation, was downregulated during dehydration treatment. These results suggest that maintaining a lower level of *CaWAKL20* expression to slow cell growth is beneficial for pepper tolerance to injury from heat stress. This is also supported by our transgenic data (Figures 3, 4) as well as the predicted interaction between CaWAKL20 and the growth regulator EGR2 (Supplementary Figure S1D), although the functional mechanisms involved require further detailed study.

Abscisic acid plays important roles in plant responses to a range of environmental stresses, including heat stress. ABA is thought to perform a number of cellular functions, such as controlling the production of protective enzymes and regulating the transfer of water, to keep plant cells alive from heat stress (reviewed by Vishwakarma et al., 2017). Heat acclimation triggers an increase in endogenous ABA content (Liu et al., 2006); conversely, exogenous ABA enhances thermotolerance by upregulating the expression of heat-shock proteins (HSPs), including transcription factors (Wang et al., 2017). In this study, CaWAKL20 was predicted to interact with three ABA signaling components – ABI2, HAI1, and AHG1 (Supplementary Figure S1D) – and *CaWAKL20* expression was rapidly induced by ABA treatment (Figure 2C). Meanwhile, Arabidopsis *CaWAKL20*-OE lines showed a lower sensitivity to ABA than the EV line for seed germination, seedling survival, and root growth (Figure 5), and the enhanced thermotolerance of Arabidopsis seedlings by ABA pre-treatment was compromised in *CaWAKL20*-OE lines (Figure 6). These results suggest that *CaWAKL20* functions in an ABA-related pathway. In addition, the heat-induced expression of several ABA-responsive genes, *AREB*, *ABF*, *DREB*, and *HSFA3*, and some key regulator genes for plant thermotolerance, *HSFA1a*, *HSFA2*, and *HSFA7a*, was reduced in Arabidopsis *CaWAKL20*-OE lines compared to the EV line (Figure 7). Interestingly, similar phenomena were observed in the plastid casein kinase 2 (CK2) knockout mutant; CK2 is a major serine/threonine-specific kinase in the chloroplast stroma, and the authors argued that CK2 positively regulated retrograde signaling from the plastid to the nucleus during plant responses to ABA and heat stress (Wang et al., 2014). These results indicate that CaWAKL20 negatively modulates pepper plant thermotolerance by repressing the expression of ABA-responsive genes.

As universal signals, ROS might integrate with hormone signaling, including ABA signaling, to tailor the cellular homeostasis under stress conditions (Suzuki and Katano, 2018). In our study, the expression was also induced by exogenous H_2_O_2_ with a pattern similar to that of ABA (Figure 2D). Suzuki et al. (2013) reported that ROS was responsible for the spread of heat signal from the initial site to the entire plant, and ABA specifically regulated plant acclimation to heat stress. Thus, it can be hypothesized that *CaWAKL20* functions to link the ROS signal and ABA pathway. In this model, both ABA and H_2_O_2_ induce

the expression of *CaWAKL20*, and *CaWAKL20* expression further negatively regulates ABA signaling by decreasing the ABA sensitivity of plant growth and inhibiting the induced-expression of ABA-responsive genes and key regulator genes for thermotolerance under heat stress. However, which node in the ABA signal pathway, such as perception, biosynthesis, degradation, or signaling, is regulated by CaWAKL20 needs more investigation to verify.

## Conclusion

In this study, we report that pepper CaWAKL20 possesses the conserved domains of the WAK/WAKL family and is localized to the plasma membrane and linked to the cell wall. The expression of *CaWAKL20* was downregulated by heat stress but upregulated by ABA treatment. Silencing *CaWAKL20* expression enhanced pepper thermotolerance, but *CaWAKL20* overexpression decreased Arabidopsis tolerance to heat stress. In addition, overexpressing *CaWAKL20* reduced Arabidopsis sensitivity to ABA and decreased the heat-induced expression of ABA-responsive genes. Therefore, we suggest that *CaWAKL20* negatively modulates plant thermotolerance by inhibiting ABA-responsive gene expression. Our results lay a foundation for further understanding of the functional mechanisms of WAKs/WAKLs during plant adaptation to environmental stress.

## Author Contributions

HW and MLu designed the research. HW, HN, MLi, YZ, and WH performed the experiments. HW and HN analyzed the data and drafted the manuscript. QD, YD, and MLu revised the manuscript and contributed reagents, materials, and analysis tools.

## Conflict of Interest Statement

The authors declare that the research was conducted in the absence of any commercial or financial relationships that could be construed as a potential conflict of interest.

## Footnotes

1 ftp://ftp.solgenomics.net/genomes/Capsicum_annuum/C.annuum_zunla/annotation/

2 https://www.arabidopsis.org/

3 http://smart.embl-heidelberg.de/

4 http://www.ebi.ac.uk/Tools/msa/clustalo/

5 http://string-db.org

6 https://www.ncbi.nlm.nih.gov/tools/primer-blast/

7 http://vigs.solgenomics.net/
