# Supplementary figures and images for: A Wall-Associated Kinase Gene CaWAKL20 From Pepper Negatively Modulates Plant Thermotolerance by Reducing the Expression of ABA-Responsive Genes
Source: Front Plant Sci. 2019 May 14;10:591. doi: 10.3389/fpls.2019.00591 (PMC6528620; doi:10.3389/fpls.2019.00591)

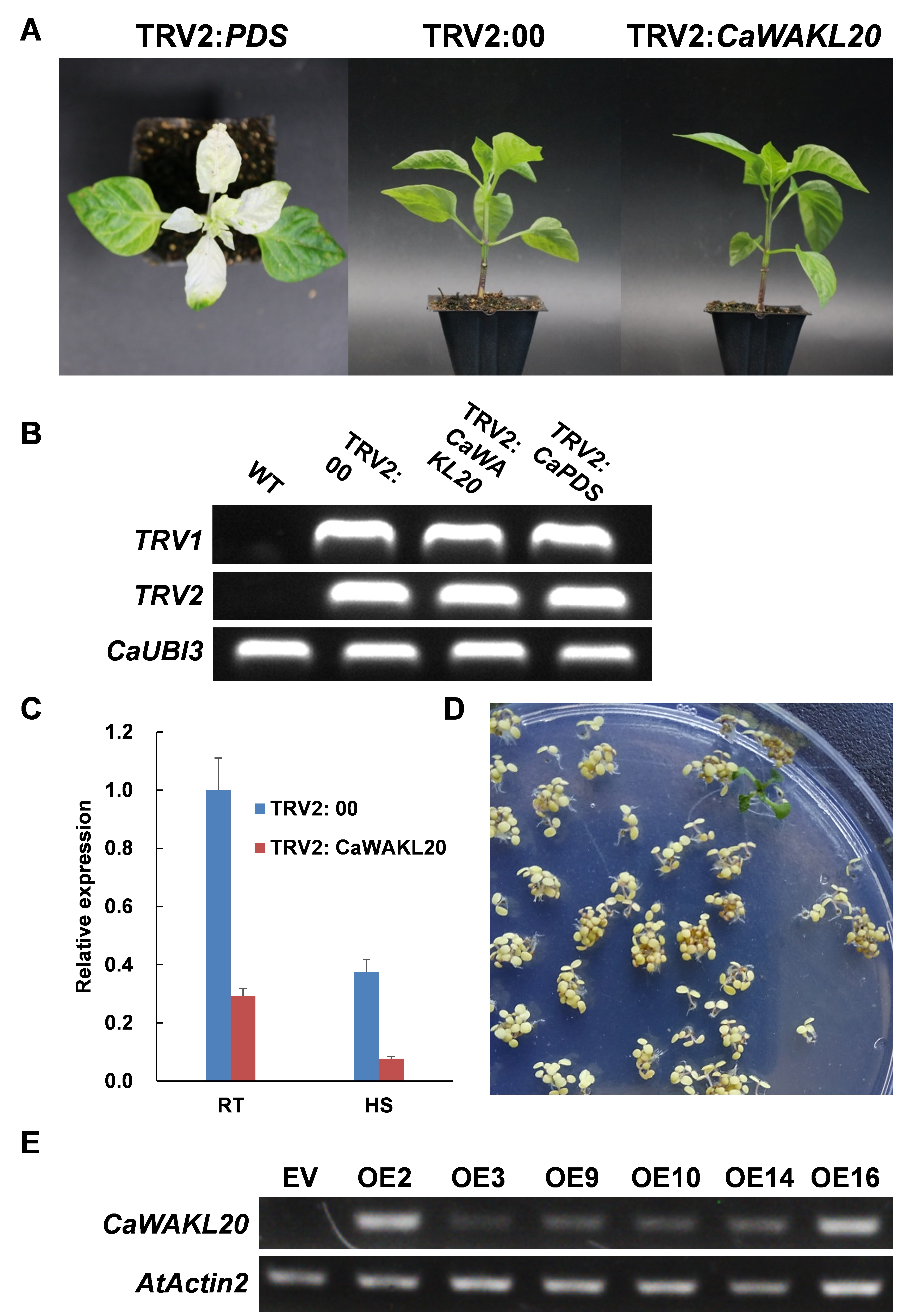

Supplement: Supplementary file 2 [file Image_2.JPEG]
