# Supplementary material for: A Wall-Associated Kinase Gene CaWAKL20 From Pepper Negatively Modulates Plant Thermotolerance by Reducing the Expression of ABA-Responsive Genes
Source: Front Plant Sci. 2019 May 14;10:591. doi: 10.3389/fpls.2019.00591 (PMC6528620; doi:10.3389/fpls.2019.00591)
Supplement: Supplementary file 3 [file Data_Sheet_1.docx]

**SUPPLEMENTARY MATERIALS**

**Figure S1** Analysis of the deduced *CaWAKL20* amino acid sequence. (**A, B**) Conserved domains in CaWAKL20. Red rectangle, signal peptide; blue rectangle, transmembrane region; EGF, epidermal growth factor; EGF-CA, calcium-binding EGF-like domain; GUB-WAK_bind domain (pfam13947), cysteine-rich galacturonan-binding domain; Pkinase domain (pfam00069), serine/threonine protein kinases and tyrosine protein kinases; S_TKc domain (cd14066), serine/threonine protein kinases, catalytic domain. (**C**) Phylogenetic tree of CaWAKL20 with 26 WAK/WAKL members from Arabidopsis. The number presents the bootstrap value. (**D**) Predicted CaWAKL20 protein-protein interaction network. PP2C, protein phosphatase 2C; PP2C74, Capana10g001593 (At5g36250); PP2C76, Capana07g002353 (At5g53140); PP2C80, Capana01g003330 (At5g66720); ABI2 (ABA insensitive 2), Capana08g000504 (At5g57050); AHG1 (ABA-Hypersensitive Germination 1), Capana05g002193 (At5g51760); APD9 (Arabidopsis PP2C clade D9), Capana02g002511 (At5g66080); EGR2 (E Growth-Regulating 2), Capana08g001566 (At5g27930); HAI1 (Highly ABA-Induced PP2C gene 1), Capana05g002193 (At5g59220); WAKL7, Capana11g001520 (At1g16090); Zinc ion binding protein, Capana11g001824 (At5g57820).

**Figure S2** Confirmation of *CaWAKL20*-silenced pepper seedlings and *CaWAKL20*-overexpressing Arabidopsis lines. (**A**) Phenotypes, (**B**) TRV gene expression and (**C**) efficiency of gene expression silencing in *CaWAKL20*-silenced pepper seedlings 30 d after inoculation. The *CaUBI3* gene was used as a reference. *PDS*, phytoene desaturase gene for chlorophyll synthesis; TRV2:00, control seedlings with the empty TRV2 vector; TRV2:*CaWAKL20*, *CaWAKL20*-silenced seedlings. RT, room temperature; HS, heat stress induction at 45°C for 1 h. Error bars represent standard deviations from three biological replicates. (**D**) Screening with kanamycin in MS medium and (**E**) determination of gene expression levels in *CaWAKL20*-overexpressing Arabidopsis lines. EV, control transgenic Arabidopsis line with the empty vector; OE, Arabidopsis transgenic lines with *CaWAKL20*. The *AtActin2* gene was used as the internal control.
