# Supplementary material for: A Wall-Associated Kinase Gene CaWAKL20 From Pepper Negatively Modulates Plant Thermotolerance by Reducing the Expression of ABA-Responsive Genes
Source: Front Plant Sci. 2019 May 14;10:591. doi: 10.3389/fpls.2019.00591 (PMC6528620; doi:10.3389/fpls.2019.00591)
Supplement: Supplementary file 4 [file Table_1.docx]

**Table S1 Primers used in this study**

| **Primers** | **Sequence (5'–3')** |
| --- | --- |
| cDNA-CaWAKL20-F | TCATTTTCCATAGCAGCCCTG |
| cDNA-CaWAKL20-R | TCTCACTTTCTGGCTTTCACTCC |
| qCaWAKL20-F | AGGTGTTTCTGTCATTCTGGG |
| qCaWAKL20-R | GTGCATCCTTAATTCGCTTG |
| qCaUBI3-F | TGTCCATCTGCTCTCTGTTG |
| qCaUBI3-R | CACCCCAAGCACAATAAGAC |
| TRV2-CaWAKL20-F | TTCTTGGTGCTGGGGGTTATG |
| TRV2-CaWAKL20-R | CGATGGTAAATGGGAGGAACTG |
| GFP-CaWAKL20-F | AACTCCAAGCAAAAGAAATC |
| GFP-CaWAKL20-R | TTCTATTGGCTTTGCAGTAT |
| TRV1-TL-F | ATTGAGGCGAAGTACGATGG |
| TRV1-TL-R | GTAAAATCATTGATAACAACACAGACAAAC |
| TRV2-Coat P-F | CGGGCTAACAGTGCTCTTG |
| TRV2-Coat P-R | CTCCCTTGGTTCGTCGTAAC |
| qAtActin2-F | TGTGCCAATCTACGAGGGTTT |
| qAtActin2-R | TTTCCCGCTCTGCTGTTGT |
| qAtABF3-F | CTGATACAGACGCAGGAGAGG |
| qAtABF3-R | AGGAACAGGGGACAAAGATG |
| qAtHSFA3-F | GAAGTTCATCAAGCACCACCA |
| qAtHSFA3-R | GGATACATCAAGTTCTTGCCTTTT |
| qAtAREB1-F | GAGAGAAGGCAAAGGAGAATGA |
| qAtAREB1-R | CTTCAAGCTCCACGGTGTAAG |
| qAtDREB2A-F | GACCTAAATGGCGACGATGT |
| qAtDREB2A-R | TCGAGCTGAAACGGAGGTAT |
| qAtHSFA6b-F | GTGATGAAAGTGGTTATGGGAATG |
| qAtHSFA6b-R | TCCGACATCTCGAATTCAGACAT |
| qAtHsfA1a-F | CCGGAGTTTTCTCGTGATCT |
| qAtHsfA1a-R | TCGATCTGGATCCACTTTCC |
| qAtHsfA2-F | TGGGATTCTCATAAGTTCTCAACA |
| qAtHsfA2-R | TGGATCAATCTTTCTGAATCCAT |
| qatHsfa7a-F | GGGTTTACACGAAAATGCTCC |
| qatHsfa7a-R | GAATGCAAATCCCAGACGAC |
